# “Pain Relief is an Essential Human Right”, We Should be Concerned about It

**DOI:** 10.5812/kowsar.22287523.2306

**Published:** 2011-09-26

**Authors:** Farnad Imani, Saeid Safari

**Affiliations:** 1 Department of Anesthesiology and Pain Medicine, Tehran University of Medical Sciences, Tehran, Iran

**Keywords:** Anesthesia and analgesia, Pain, Illness burden, Pain clinics, Regional anesthesia

Pain is a major public health issue throughout the world and represents a major clinical, social, and economic problem (1). The clinical, social, and economic costs of chronic pain in added health care costs, lost productivity, and lost income are significant, and if prolonged, it can cause distress, anxiety, and suffering. The burden that pain can place on individuals and the huge costs that society must bear as a result clearly indicate the need for collective thinking through a decision-making process (2). Acute pain is a major challenge worldwide, and chronic pain poses a massive disease burden, affecting an estimated 20% of adults, rising to 50% of the older population. In addition, cancer-related pain affects 70% of the 10 million cancer patients who are diagnosed annually, which is expected to double by 2020 (3).

On October 11, 2004, the Global Day Against Pain, access to pain relief was promoted as an essential human right by the IASP, WHO, and European Federation of IASP Chapters (EFIC) (4, 5). Human rights refer to the concept of a universal right, regardless of legal jurisdiction or other localizing factors, such as ethnicity, nationality, and sex. The UN Universal Declaration of Human Rights conceptualizes human rights as based on inherent human dignity (3). Documents that were released at that time demonstrated that pain control has been a neglected area of governmental concern (6). There is a large and widening gap between the increasingly sophisticated knowledge of pain and its treatment and the effective application of that knowledge (7). Although the incidence of pain in developing countries is higher and cost-effective methods for pain care are available, acute and chronic pain is undertreated, and timely access to care is a growing problem in nations with access to the best health care (2, 8).

Acute and chronic pain are often poorly managed for a wide variety of cultural, political, attitude-related, educational, and logistical reasons (8). Under treatment of pain is a poor medical practice that results in many adverse effects (7). Improvements in clinical pain care have not matched advances in scientific knowledge, and innovations in medical education on pain are needed. Several lines of evidence indicate that pain education needs to address the affective and cognitive dimensions of pain (9).

The practice of pain medicine is affected by many market forces, including industry relationships with pain providers, lawmakers, and insurance companies; direct to consumer advertising; insurance reimbursement patterns; and competition among health care systems and pain management providers (10). These economic factors can encourage innovation and efficiency and may increase access to pain treatment.

The Board of Directors of the American Board of Pain Medicine defined the specialty of pain medicine as follows: “the specialty of pain medicine is concerned with the prevention, evaluation, diagnosis, treatment and rehabilitation of painful disorders”. Interventional pain management is defined officially by National Uniform Claim Committee (NUCC) as “the discipline of medicine devoted to the diagnosis and treatment of pain and related disorders by the application of interventional techniques in managing subacute, chronic, persistent, and intractable pain, independently or in conjunction with other modalities of treatments”(11).

Whereas anesthesiologists constitute the majority of physicians who treat chronic pain, other specialties, including psychiatry, physical medicine and rehabilitation, neurology, neurosurgery, and primary care (not mutually exclusive), are also heavily involved with chronic pain management. Development of pain medicine as a separate specialty does not prevent other specialties from managing pain syndromes or developing a multidisciplinary approach to pain management. The benefit of pain management by other disciplines is the appropriate and timely referral of these patients to pain specialists, which ultimately may be helpful to the patient (11). True pain practitioners stand ready, able, and willing to perform a comprehensive assessment, guide complex diagnostic evaluation, and offer a broad range of treatment options to patients with chronic and cancer-related pain. The growing tendency among Iranian anesthesiologists toward regional anesthesia and pain medicine in recent years spurred the establishment of the Iranian Society of Regional Anesthesia and Pain Medicine (ISRAPM) in November 2006 improve and support scientific and educational activities in this field, with the following goals:

- To standardize the indications, approaches, and techniques for regional anesthesia and pain interventions
- To maximize the exposure, education, and training of pain fellows
- To advance patient safety, cost effectiveness, and accountability
- To exchange and share new information, ideas, and innovations concerning regional anesthesia and pain management
- To encourage basic science as well as clinical outcome research in this field
- To promote information on regional anesthesia and pain procedures
- To preserve coverage for regional anesthesia and interventional pain management
- To encourage specialization and research in these areas
- To encourage the teaching of regional anesthesia and interventional pain procedures in all anesthesiology training programs

Since 2006, ISRAPM stepped toward relieving pain by training pain medicine fellowships and conducting annual international ISRAPM seminars in interventional pain management. Considering the progressive interest in research projects on pain medicine, the lack of scientific journals that cover and share creative and innovative materials and articles was highlighted specially in Middle East and Asia. With the aim of disseminating updates on pain medicine and interventional pain, ISRAPM has published “Anesthesiology and Pain Medicine” since the summer of 2011.

Anesthesiology and Pain Medicine is the official Journal of ISRAPM, covering clinical and basic research, education, patient care, health economics, and policy to inform all practitioners in pain management, such as anesthesiologists, interventional pain physicians, neurosurgeons, neurologists, and any specialists who are interested in pain medicine (12). The 4th national and 2nd International ISRAPM Congress will be held on October 28–30, 2011 in Tehran, Iran. Noting the requirement of wider sources of exchanging information, sharing data, and networking among researchers, ISRAPM aims to bolster networking between pain physicians and those who care about pain by holding workshops and speeches on interventional pain management and cutting-edge research locally, nationally, and internationally.

On behalf of scientific and organizing committees, we are honored to invite you to join the ISRAPM Congress 2011: Interventional Pain Medicine, This meeting will address many of the issues facing us as medical practitioners who treat patients with various types of pain. Aspects of pain that will be addressed include neuropathic pain, back pain, cancer pain, palliative care, and acute pain management. Attention will be given to both the theory and practice of modern interventional pain treatment. The meeting will include lectures, workshops, and face to face “meet the expert” sessions. We hope to provide all our colleagues and friends with the opportunity to encounter new findings and technologies. We sincerely anticipate making the congress as a meeting point where all our colleagues and friends gather and exchange their latest knowledge and experiences in their fields.
